# 3D Self-Localization and Tracking with Minimum Anchor Dependency: A Hybrid Measurement and EKF-Based Approach

**DOI:** 10.3390/s26123925

**Published:** 2026-06-20

**Authors:** Amani Atiani, Mohammed El-Absi, Thomas Kaiser

**Affiliations:** Institute of Digital Signal Processing, University of Duisburg-Essen, 47057 Duisburg, Germany; mohammed.el-absi@uni-due.de (M.E.-A.); thomas.kaiser@uni-due.de (T.K.)

**Keywords:** terahertz, indoor self-localization and tracking, infrastructure dependency, sub-mm accuracy, extended Kalman filter, Fisher information matrix, hybrid scheme

## Abstract

This paper investigates the feasibility of 3D self-localization and tracking using chipless radio frequency identification (RFID) tags operating in the terahertz (THz) frequency band. The primary objective is to achieve sub-millimeter (sub-mm) localization and tracking accuracy while minimizing reliance on external infrastructure. To this end, a hybrid localization framework is proposed that jointly exploits round-trip time-of-flight (RToF) and angle-of-arrival (AoA) measurements to enhance localization performance. Although near-field propagation effects are inherently significant in the considered THz operating regime, a simplified far-field approximation is adopted to facilitate tractable system modeling and analytical development. The proposed framework is further extended to dynamic scenarios through an extended Kalman filter (EKF)-based tracking algorithm, which incorporates temporal state evolution to improve estimation robustness under noisy measurements. Furthermore, the Cramér–Rao lower bound (CRLB) for the hybrid RToF-AoA system is derived to establish the fundamental limits of localization accuracy under varying system configurations and measurement conditions. Simulation results demonstrate that the proposed approach is capable of achieving sub-mm localization and tracking accuracy with a highly constrained anchor infrastructure, including operation with a single anchor in the considered scenario. These findings highlight the potential of THz chipless RFID technology as a promising enabling solution for next-generation high-accuracy localization and tracking applications.

## 1. Introduction

Localization has emerged as a key enabling technology for sixth-generation (6G) wireless networks, supporting applications that require highly accurate and real-time position information [1,2]. Conventional radio-frequency (RF) localization techniques operating at lower frequencies are inherently constrained by their long wavelengths, limited bandwidth, and susceptibility to multipath degradation. In contrast, terahertz (THz) technology is attractive because of its extremely short wavelength and ultra-wide bandwidth, which enable the compact integration of large-scale antenna arrays for high-resolution angle-of-arrival (AoA) estimation and precise time-of-flight (ToF) measurement, even in complex propagation environments [1,3].

In indoor scenarios, where global navigation satellite systems (GNSS) are unavailable and multipath and shadowing are severe, THz-based localization becomes increasingly important for emerging 6G applications such as robotic manipulation, autonomous navigation, synthetic aperture radar (SAR) imaging for non-destructive testing, collaborative industrial systems, extended reality (XR) environments, and digital-twin implementations that demand centimeter (cm) to sub-millimeter (sub-mm) level localization accuracy [4]. Self-localization systems represent a paradigm shift from conventional localization frameworks, enabling mobile agents to determine their position autonomously without relying on dense infrastructure or external reference networks. Among these approaches, simultaneous localization and mapping (SLAM) has become a key tool in autonomous robotics, allowing agents to build maps of unknown environments while simultaneously estimating their own position [5,6]. SLAM is widely used in robotic and inspection applications [7]. However, achieving sub-mm accuracy using SLAM alone remains highly challenging, especially in THz sensing scenarios that demand extremely high localization accuracy [8].

### 1.1. Problem Statement

In our previous work [9], we demonstrated that SLAM alone cannot achieve sub-mm localization accuracy without additional supporting infrastructure. To overcome this limitation, we subsequently proposed a self-localization framework assisted by multiple fixed low-cost chipless radio frequency identification (RFID) tags [10]. The integration of this framework with indoor SAR imaging was then investigated experimentally in [11,12], where the feasibility of sub-mm localization was confirmed. The large bandwidth available at THz frequencies provides fine delay resolution and highly accurate ranging, making it particularly attractive for precise localization and tracking. Exploiting these properties, our chipless RFID-assisted self-localization approach achieved sub-mm accuracy at THz frequencies [10], while the underlying concept was later validated experimentally at lower GHz frequencies in [13]. To further improve the practicality of the system, we investigated optimized chipless RFID tag deployment strategies in [14], reducing the number of required anchors while maintaining localization coverage. Despite these advances, our previous studies reveal that achieving sub-mm accuracy still requires a relatively dense infrastructure of fixed reference tags. Consequently, although THz systems inherently support very high localization precision, the attainable accuracy remains strongly coupled to anchor density, leading to a fundamental trade-off between infrastructure deployment and localization performance. Addressing this limitation constitutes the main motivation of the present work. Building upon our earlier RFID-assisted self-localization framework, we develop an indoor self-localization and tracking system that reduces infrastructure dependency while preserving sub-mm localization accuracy and enabling reliable tracking of moving targets. Since the proposed system operates in the THz band, near-field propagation effects become significant, and the curvature of the wavefront must be explicitly incorporated into the localization and tracking model.

### 1.2. Related Work

Several studies have investigated localization and tracking in 5G and beyond, including model-based, reconfigurable intelligent surface (RIS)-assisted, artificial intelligence (AI)-driven, and RFID-based approaches. Model-based low-infrastructure methods have shown that localization can be achieved with limited anchor support. For example, ref. [15] estimates 2D position and 1D orientation using a single 5G mmWave transmitter under both line-of-sight (LoS) and non-line-of-sight (NLoS) conditions. A Bayesian-filter-based vehicular SLAM method at mmWave is presented in [16], while [17] employs an extended Kalman filter (EKF) for joint position and orientation tracking using a lens antenna array and a single base station.

A 3D map-assisted method in [18] exploits multi-bounce reflections, AoA, ToF, and environmental maps, achieving sub-meter accuracy at mmWave and cm-level accuracy at sub-THz with only one base station. RIS-enabled methods provide another important direction for self-localization with reduced infrastructure. A 3D self-localization system for a fixed target using a single RIS at mmWave is introduced in [19]. Likewise, ref. [20] proposes a RIS-enabled self-localization framework without access points at 28 GHz, achieving sub-mm near-field accuracy. The feasibility of RIS-enabled 2D self-localization without access points is further validated experimentally in [21] using 60 GHz frequency modulated continuous wave radar.

Localization under a near-field propagation model offers a distinct advantage, as the additional spatial information conveyed by wavefront curvature can improve positioning accuracy and reduce dependence on a large number of anchors, albeit at the expense of increased algorithmic complexity [22]. Although the development of an exact near-field localization and tracking framework could potentially outperform the approximate approach considered in this work, such an investigation is beyond the scope of this paper.

The near-field regime is especially relevant for large-scale antenna arrays, RIS, and short-range links, where far-field approximations are often used to reduce complexity. In [22], a uniform linear array (ULA) is partitioned into non-overlapping sub-arrays processed with conventional far-field estimators, while wavefront curvature information improves parameter identifiability through a full row-rank Fisher Information Matrix (FIM). Similar sub-array approaches have been adopted in RIS-assisted near-field localization [23,24,25,26], where the RIS is divided into sub-surfaces satisfying the far-field assumption.

Array-of-sub-arrays (AoSA) architectures are also widely used in THz systems due to their implementation and signal processing benefits [1]. Recent works further investigate optimal sub-array configurations, highlighting the trade-off between localization performance and computational complexity.

AI- and machine learning-based localization methods have also received increasing attention, particularly for indoor scenarios. Examples include unified indoor–outdoor localization combining model-based geometry and unsupervised learning [27], deep learning-based 3D indoor localization [28], and model-driven machine learning with data augmentation for sub-wavelength accuracy [29]. Although such methods can achieve competitive accuracy, they often require substantial training data and increased computational complexity. To mitigate this, ref. [30] proposes a compact neural network for learning the relationship between angle-difference-of-arrival and target location. In the THz domain, ref. [31] introduces a structured bidirectional long short-term memory for indoor 3D NLoS localization, while ref. [32] shows that deep learning can also be effective for model-based multi-target tracking.

Additional related works address deployment and practical localization systems. The authors of [33] demonstrated a 3D indoor positioning and orientation estimation approach based on fast-chirp frequency-modulated continuous-wave radar, and assisted with identifiable Doppler tags. The system operates at 77 GHz, with tags positioned within a short range of the radar. Four tags are required to determine the position, whereas orientation can be estimated using two tags. Reference-node deployment strategies for LoS coverage in indoor RF/optical wireless communication networks are studied in [34]. A trilateration-based photonic THz radar using three corner reflectors is presented in [35], achieving a maximum positioning error of 3.2 mm over 0.6 m. In RFID-based localization, ref. [36] introduces a portable robot-mounted RFID locator with an average error of 6 cm within 1 m, while ref. [37] presents a 2D self-localization system for mobile robots that fuses ultra high frequency RFID phase measurements with odometry using multiple unscented Kalman filters, achieving a 2.7 cm root mean squared error (RMSE). Despite these advances, prior work has not addressed 3D THz self-localization and tracking of a mobile robot with an explicit emphasis on minimizing infrastructure dependency while targeting sub-mm accuracy. This gap motivates the present work.

### 1.3. Motivation and Contributions

This paper aims to achieve sub-mm localization and tracking accuracy with minimal infrastructure. The central idea is that hybrid distance–angle measurements and temporal tracking can reduce anchor requirements while preserving high localization accuracy. In particular, hybrid schemes improve estimation accuracy by increasing measurement diversity and resolving geometric ambiguities [38,39]. Tracking further enhances performance by combining the mobility model with periodic observations, enabling accurate position estimation even when fewer measurements are available. Moreover, the considered self-localization and tracking setting is monostatic. In dynamic operation, it can also exploit prior state information, which further reduces infrastructure requirements and estimation complexity [40]. These properties motivate a self-localization and tracking framework with explicitly reduced anchor dependency.

However, studies on 3D indoor self-localization and tracking at THz are limited, and sub-mm accuracy is still largely unexplored. Moreover, none of the aforementioned works explicitly treat infrastructure dependency as a design constraint. This work extends our previous conference study in [41] to a 3D setting by employing a robot-mounted antenna array that exploits AoA in addition to range measurements. The impacts of anchor geometry and measurement accuracy are also investigated. Here, “infrastructure” refers to the pre-installed chipless RFID tags deployed in the environment. With the adopted hybrid measurement model, each anchor provides three measurements: RToF and AoA measurements in azimuth and elevation. This substantially reduces the anchor requirement and can enable single-anchor 3D position estimation under the assumptions used in this paper.

The main contributions of this work are summarized as follows:

- We propose a robust 3D THz self-localization and tracking framework based on hybrid measurements and minimal anchor infrastructure, targeting sub-millimeter positioning accuracy. The 3D snapshot localization problem is formulated as a nonlinear least-squares (NLLS) estimation problem, with near-field propagation modeled through a piecewise far-field approximation.
- We develop a comprehensive theoretical framework for both localization and tracking by deriving the corresponding FIM for snapshot localization and extending the proposed approach to dynamic scenarios using an EKF. These theoretical performance bounds are established to characterize the achievable estimation accuracy.
- We investigate the fundamental trade-off between measurement accuracy and the number of available observations and conduct extensive simulation studies to evaluate the impact of measurement quality, mobility, and anchor geometry on sub-mm localization and tracking performance, providing insights into both theoretical limits and practical system design.

The remainder of this paper is organized as follows. Section 2 presents the system model and the self-localization and tracking setup. Section 3 introduces the 3D hybrid-based self-localization estimator based on NLLS, and derives the corresponding FIM, with emphasis on infrastructure dependency. Section 4 presents the 3D self-tracking algorithm. Section 5 reports the simulation results and discussion. Finally, Section 6 concludes the paper.

Symbols and Notations: Bold lowercase letters denote vectors (e.g., x), while bold uppercase letters denote matrices (e.g., X). Italic lowercase letters denote scalars (e.g., *x*). Furthermore, $∥\cdot ∥$, ${\left(\cdot \right)}^{T}$, ${\left(\cdot \right)}^{-1}$, and $\mathrm{tr}\;\left[\cdot \right]$ represent the $l_{2}$ norm, transpose, inverse, and trace, respectively. Moreover, diag[a] denotes a square diagonal matrix with the elements of vector a on its main diagonal, and $\mathrm{blkdiag}\;\left[\cdot \right]$ denotes a block-diagonal concatenation of input matrices. The operator $\left(\frac{\partial }{\partial p}\right)$ denotes the partial derivative with respect to p, and $I_{M}$ denotes the identity matrix of size *M*. Finally, δ(t) denotes the Kronecker delta function, while ⪰ and ≻ denote the positive semidefinite and positive definite relations, respectively.

## 2. System Model for Self-Localization and Tracking

Object localization refers to estimating its spatial state from a set of observations, where the observations are nonlinear functions of the object location. This corresponds to an indirect, or multistage, localization process, in contrast to direct localization [1]. In the considered self-localization setting, the robot is initially unaware of its own position and must estimate it. When the robot is moving, this task naturally extends to self-tracking.

### 2.1. System Configuration

We consider an indoor chipless RFID-based self-localization and tracking system operating in the THz band. The robot moves along a nonlinear 3D trajectory and is equipped with an RFID reader comprising a uniform planar antenna array (UPA) with $N=N_{a}\times N_{b}$ elements. To approximate the near-field model, the UPA is divided into non-overlapping sub-UPAs [22], denoted by ${\tilde{N}}_{1},{\tilde{N}}_{2},...,{\tilde{N}}_{U}$, where *U* is the number of the non-overlapping sub-UPAs, and ${\tilde{N}}_{u}={\tilde{N}}_{ua}\times {\tilde{N}}_{ub}$ for u=1,2,3,...,U. Using a greater number of smaller sub-UPAs yields a more accurate approximation, albeit with reduced AoA resolution. Conversely, employing fewer, larger sub-UPAs provides higher AoA resolution but a coarser approximation. The robot is assumed to be initially unaware of its position, and all localization-related signals and data processing are carried out at the reader.

**Figure 1:**
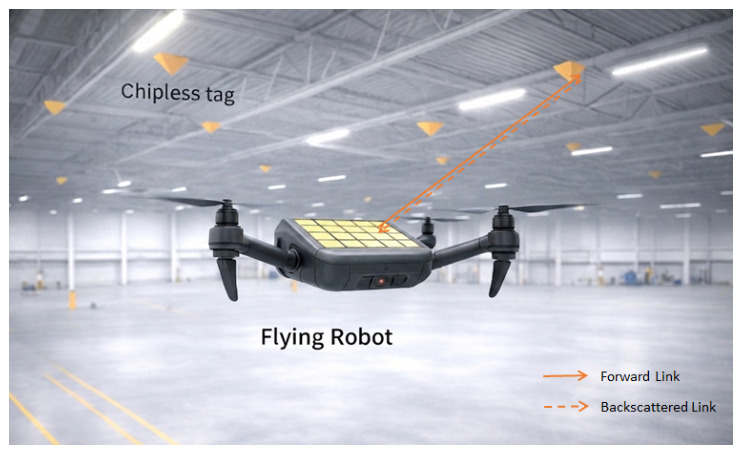
The system setup (enhanced by Copilot 2026).

To perform self-localization and tracking, the received signals from fixed anchors are used to estimate the robot’s location. This estimation problem arises from the inherently nonlinear relationship between the anchor positions and the target’s coordinates. The anchors are passive chipless tags as described in [10,42,43]. The *m*th tag is located at the fixed position $p^{\left(m\right)}={\left[p_{x}^{\left(m\right)},p_{y}^{\left(m\right)},p_{z}^{\left(m\right)}\right]}^{T}\in R^{3}$, for m=1,2,...,M, where *M* denotes the number of tags. The tag positions are pre-determined using the placement optimization algorithm described in [14], which is used to ensure k-coverage throughout the 3D environment, i.e., ensuring that each robot location in the 3D environment is covered by at least *k* reference tags. For range-only 3D localization, at least four anchors, i.e., k=4, are required. Incorporating AoA measurements can reduce the anchor requirement, and the hybrid RToF–AoA formulation can, under the assumptions adopted in this paper, enable single-anchor 3D position estimation. The considered system configuration is illustrated in Figure 1.

### 2.2. System and State Models

In the dynamic case, tracking extends snapshot localization by combining periodic measurements with a motion model to improve state estimation. The dynamic estimation problem is modeled in a standard nonlinear state-space form, where f(.) and g(.) denote the state-transition and measurement functions, respectively. At each epoch *k*, the model can be written as [5]

(1) $$\begin{array}{c} x_{k}=f\left(u_{k},x_{k-1}\right)+w_{k}, \end{array}$$

(2) $$\begin{array}{c} z_{k}=g\left(x_{k}\right)+\nu_{k}. \end{array}$$

Here, $x_{k}$ denotes the state vector, and $z_{k}$ is the measurement vector. Its dimension and elements are determined by the number and types of measurements considered, as presented in Section 3, and $u_{k}$ is the control input. The process noise $w_{k}∼N\left(0,Q_{k}\right)$ and the measurement noise $\nu_{k}∼N\left(0,R_{k}\right)$ are assumed to be mutually independent Gaussian random vectors with covariance matrices $Q_{k}$ and $R_{k}$, respectively. The estimator is initialized with the state estimate $x_{0}$ and an associated error covariance matrix $P_{0}$. In the static case, when $x_{k}=x_{k-1}$, the dynamic tracking problem reduces to snapshot localization.

The robot motion is modeled through the nonlinear state-transition function $f(u_{k},x_{k-1})$ in (1). At epoch *k*, the unknown robot state is defined as $x_{k}={\left[{p_{k}}^{T},\xi_{k},\psi_{k}\right]}^{T}\in R^{5}$, where $p_{k}={\left[x_{k},y_{k},z_{k}\right]}^{T}\in R^{3}$ represents the 3D position vector in meters, and $\xi_{k}\in \left(-\pi ,\pi \right)$, and $\psi_{k}\in \left(-\frac{\pi }{2},\frac{\pi }{2}\right)$ denote the heading (pitch) and yaw orientations in radians, respectively. The orientation variables are included in the dynamic state to support the motion and measurement models. In the snapshot self-localization stage developed in Section 3, the estimation focuses on the 3D position $p_{k}$, while $\xi_{k}$ and $\psi_{k}$ are assumed to be available from onboard orientation sensing or prior state information.

A common motion model for mobile robots is the constant-turn-rate-and-velocity (CTRV) model, given by [5]:

(3) $$\begin{array}{c} x_{k}=x_{k-1}+\left[\begin{array}{c} -\frac{v}{\omega }\cos\psi_{k-1}\left[\sin\xi_{k-1}-\sin\left(\xi_{k-1}+\omega dt\right)\right]\\ \frac{v}{\omega }\cos\psi_{k-1}\left[\cos\xi_{k-1}-\cos\left(\xi_{k-1}+\omega dt\right)\right]\\ vdt\sin\psi_{k-1}\\ \omega dt\\ 0 \end{array}\right]+w_{k}. \end{array}$$

Here, dt denotes the epoch duration in seconds, and $u_{k}={\left[v,\omega \right]}^{T}\in R^{2}$ is the control input, where *v* and ω are the translational velocity and rotational velocity, respectively. These quantities are assumed to be available from onboard odometry or motion sensing and are therefore treated as known exogenous inputs. Hence, in the considered model, *v* and ω are assumed to be constant over each sampling interval. Because the robot estimates its state recursively over time, prior state information is naturally incorporated into the motion model. This can reduce prediction uncertainty compared with approaches that do not exploit temporal state evolution [40]. The present state model is intentionally minimal. Extensions to higher-dimensional orientation states or explicit velocity-state modeling are possible but are beyond the scope of this paper.

### 2.3. Channel Model

The RFID channel is a backscattered channel consisting of two links: a forward link from the reader to the tag and a backward link from the tag to the reader. For each *u*th sub-UPA, the channel model can be considered as far-field. Consequently, at epoch *k*, the THz backward-link impulse response vector between the $m^{th}$ anchor and the *u*th sub-UPA on the robot is modeled as [10]

(4) $$\begin{array}{c} {\tilde{h}}_{k,u}^{\left(m\right)}\left(t\right)=\sum_{\tilde{l}=0}^{{\tilde{L}}_{k,u}^{\left(m\right)}-1}{\tilde{\alpha }}_{k,l,u}^{\left(m\right)}a\left({\tilde{\theta }}_{k,l,u}^{\left(m\right)}\right)\delta \left(t-\frac{{\tilde{\tau }}_{k,l,u}^{\left(m\right)}}{2}\right), \end{array}$$

where ${\tilde{L}}_{k,u}^{\left(m\right)}$ is the number of resolvable paths ($\tilde{l}=0$ is the LoS path), ${\tilde{\alpha }}_{k,l,u}^{\left(m\right)}$ is the complex channel gain, $a\left({\tilde{\theta }}_{k,l,u}^{\left(m\right)}\right)\in C^{\tilde{N}}$ denotes the array response of the reader, and ${\tilde{\tau }}_{k,l}^{\left(m\right)}$ is the RToF of the $\tilde{l}$th path from the *m*th tag. The AoA vector at epoch *k* is ${\tilde{\theta }}_{k,l,u}^{\left(m\right)}={\left[{\tilde{\theta }}_{k,l,u}^{\left(m\right)},{\tilde{\phi }}_{k,l,u}^{\left(m\right)}\right]}^{T}$ in azimuth and elevation, respectively, where $\tilde{\theta }\in R^{2}$, ${\tilde{\theta }}_{k,l,u}^{\left(m\right)}\in \left(-\pi ,\pi \right)$, and ${\tilde{\phi }}_{k,l,u}^{\left(m\right)}\in \left(-\frac{\pi }{2},\frac{\pi }{2}\right)$, and all are considered per the *u*th sub-UPA.

The forward-link impulse response has the same form as (4), except that the receive array response is replaced with the transmit array response $a^{T}\left({\tilde{\theta }}_{k,l,u}^{\left(m\right)}\right)$, and the corresponding angle is interpreted as the angle-of-departure (AoD). Under the reciprocal monostatic geometry considered here, AoA and AoD are treated equivalently; therefore, the remainder of the paper uses AoA notation. In the localization stage, the measurements associated with each anchor are assumed to be extracted from the resolved LoS path.

## 3. Hybrid Self-Localization with Reduced Infrastructure Dependency

As stated in Section 2, the snapshot localization stage estimates only the 3D position, while the orientation is assumed to be known. Hybrid localization combines multiple measurements to improve estimation accuracy and reduce geometric ambiguity. In the present work, the robot position is estimated from hybrid RToF and AoA measurements. This is particularly beneficial when the number of available anchors is limited. Although hybrid measurements improve localization accuracy, they also increase computational complexity. Let β=IM denote the total number of independent measurements, where *I* is the number of measurements contributed by each anchor. Thus, infrastructure dependency can be expressed in terms of either β or *M*.

**Figure 2:**
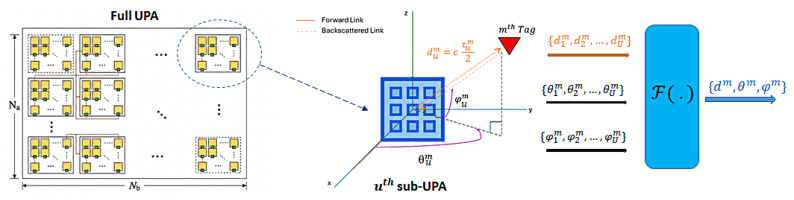
The sub-UPA approach.

By adopting the sub-UPA approach, measurements are acquired from each $u^{th}$ sub-UPA and then combined to form the final set of observations. Various combining schemes can be employed, ranging from simple averaging techniques to more advanced methods such as weighted combining. These aggregated measurements are then utilized as inputs to the localization and tracking estimators as shown in Figure 2.

Let the measurement vector at epoch *k* be expressed as $z_{k}={\left[z_{k}^{\left(1\right)},z_{k}^{\left(2\right)},...,z_{k}^{\left(M\right)}\right]}^{T}$, where $z_{k}^{\left(m\right)}$ is the observation vector associated with the *m*th anchor. Similarly, based on (2), $g\left(x_{k}\right)={\left[g^{\left(1\right)}\left(x_{k}\right),g^{\left(2\right)}\left(x_{k}\right),...,g^{\left(M\right)}\left(x_{k}\right)\right]}^{T}$, and $g^{\left(m\right)}\left(x_{k}\right)={\left[g^{\left(m,1\right)},g^{\left(m,2\right)},g^{\left(m,3\right)}\right]}^{T}$. Here, each function $g^{\left(m,i\right)}$ depends on the measurement type, with i=1,2,3, such that

(5) $$\begin{array}{c} g^{\left(m,i\right)}\left(x_{k}\right)=\left\{\begin{array}{cc} d^{\left(m\right)}\left(x_{k}\right)=∥p_{k}-p^{\left(m\right)}∥, & i=1\\ \theta^{\left(m\right)}\left(x_{k}\right)=\tan^{-1}\left(\frac{y_{k}-p_{y}^{\left(m\right)}}{x_{k}-p_{x}^{\left(m\right)}}\right)-\xi_{k}, & i=2\\ \phi^{\left(m\right)}\left(x_{k}\right)=\sin^{-1}\left(\frac{z_{k}-p_{z}^{\left(m\right)}}{∥p_{k}-p^{\left(m\right)}∥}\right)-\psi_{k}, & i=3 \end{array}\right. \end{array}$$

and,

(6) $$g^{\left(m,i\right)}\left(x_{k}\right)=F\;\left({\left\{{\tilde{g}}_{u}^{\left(m,i\right)}\left(x_{k}\right)\right\}}_{u=1}^{U}\right),$$

where F(·) denotes the combining function that fuses the set of sub-UPA measurements prior to their utilization by the localization or tracking algorithms. We adopt a two-stage localization scheme. In the first stage, RToF and AoA are estimated form the received signal. In the second stage, the robot’s location is estimated using an NLLS algorithm.

### 3.1. Channel Estimation

To estimate the robot location, the AoA $\theta_{k}^{\left(m\right)}={\left[\theta_{k}^{\left(m\right)},\phi_{k}^{\left(m\right)}\right]}^{T}$ and the RToF $\tau_{k}^{\left(m\right)}$ are first estimated independently for each anchor. Specifically, the AoA ${\tilde{\theta }}_{k,u}$ is estimated for each $u^{th}$ sub-UPA using a 2D multiple signal classification algorithm (MUSIC) over azimuth and elevation, while the RToF ${\tilde{\tau }}_{k,u}$ is estimated for each sub-UPA using a matched filter or a maximum likelihood (ML) estimator. This decoupled estimation strategy significantly reduces the search complexity compared to a 3D MUSIC approach, which jointly estimates ${\tilde{\tau }}_{k,u}^{\left(m\right)}$, ${\tilde{\theta }}_{k,u}^{\left(m\right)}$, and ${\tilde{\phi }}_{k,u}^{\left(m\right)}$. Furthermore, the sub-UPA-based formulation naturally enables parallel processing across sub-arrays, thereby avoiding additional computational overhead. More details are illustrated in Figure 2.

#### 3.1.1. RToF Estimation

Given the large bandwidth available in the THz band, the RToF-based localization is preferred for its superior time resolution, even in harsh environments [44]. Moreover, the absence of clock bias makes RToF particularly advantageous in dynamic scenarios since clock synchronization between the reader and the tag is not required [45].

In this setup, the RFID reader transmits signals to all connected tags, and each tag scatters back the received signal to the reader. The distances to the connected tags are then estimated from the RToF measurements using an ML estimator.

The estimated RToF for each connection between the robot and the *m*th anchor is ${\hat{\tau }}_{k}^{\left(m\right)}=\frac{2d_{k}^{\left(m\right)}}{c}+n_{k}^{\left(m\right)}$, where $d_{k}^{\left(m\right)}=∥p_{k}-p^{\left(m\right)}∥$ is the true distance between the target and the *m*th anchor for m=1,2,...,M, *c* is the speed of light in m/s, and $n_{k}^{\left(m\right)}$ is the measurement noise. The noise $n_{k}^{\left(m\right)}$ arises from the reader side and the tag side, which becomes more significant at THz frequencies [46].

The complexity of the ML estimator per path for *u*th sub-UPA is $O({\tilde{C}}_{u})$, where ${\tilde{C}}_{u}={\tilde{\chi }}_{t,u}{\tilde{\chi }}_{r,u}$ is the number of correlations, and ${\tilde{\chi }}_{t,u}$ and ${\tilde{\chi }}_{r,u}$ are the numbers of samples in the transmitted and received signals, respectively. Higher RToF accuracy requires more samples, which increases computational complexity. Considering *U* parallel sub-UPAs, the overall complexity of the ML estimation stage scales as $O(U{\tilde{C}}_{u})$. In addition, a combining stage is required to produce the final estimate from the *U* sub-UPA outputs, which incurs a complexity of O(U). Therefore, the total computational complexity is $O(U{\tilde{C}}_{u}+U)$, which is dominated by $O(U{\tilde{C}}_{u})$.

#### 3.1.2. AoA Estimation

Thanks to the short wavelength at THz, a large number of antenna elements can be integrated into a compact array at the reader side, i.e., on the robot. As the number of elements increases, angular resolution improves, enabling highly accurate AoA estimation and, consequently, precise localization.

In our scenario, after the signal is backscattered from the *m*th tag to the reader’s antenna array, the reader estimates the AoA using the MUSIC algorithm. Moreover, the adopted tags are assumed to provide coarse AoA estimates [42], which significantly reduces the search area, and therefore, the required computational complexity of the MUSIC algorithm. Since our goal is to achieve sub-mm localization and tracking accuracy, ultra-fine AoA estimation in both azimuth and elevation is required.

Hence, to mitigate the high complexity of the MUSIC algorithm with fine angular grids, and since the tags can provide coarse AoA estimates, we apply a multi-stage (cascaded) MUSIC algorithm instead of a one-shot MUSIC algorithm. The complexity of this hierarchical MUSIC structure per *u*th sub-UPA is approximately $O({P_{{\tilde{\theta }}_{u}}}^{\left(j\right)}{P_{{\tilde{\phi }}_{u}}}^{\left(j\right)})$ (Here, we discard the dependency of the computation’s complexity on the number of samples and the number of antennas, and include solely the effect of grid size, which is the dominant factor), where ${P_{{\tilde{\theta }}_{u}}}^{\left(j\right)}$ and ${P_{{\tilde{\phi }}_{u}}}^{\left(j\right)}$ are the grid sizes of the azimuth and elevation angles in stage *j*, respectively. In comparison, classical one-shot MUSIC has complexity $O(P_{{\tilde{\theta }}_{u}}P_{{\tilde{\phi }}_{u}})$, where ${P_{{\tilde{\theta }}_{u}}}^{\left(j\right)}\ll P_{{\tilde{\theta }}_{u}}$, and ${P_{{\tilde{\phi }}_{u}}}^{\left(j\right)}\ll P_{{\tilde{\phi }}_{u}}$. The stage-wise grid size is typically $10^{3}$ to $10^{4}$ times smaller than standard MUSIC for millidegree or sub-millidegree resolution, allowing substantial computational savings, with the accuracy trade-off depending on the refinement strategy [47].

Another improvement is the decoupled MUSIC approach, where azimuth is estimated first, selecting *S* candidate angles, followed by a narrow-range elevation search. Its complexity per sub-UPA is $O(P_{{\tilde{\theta }}_{u}}+SP_{{\tilde{\phi }}_{u}})$, where $S\ll P_{{\tilde{\theta }}_{u}}$ [48]. Both methods can be combined by using a multi-stage structure together with a decoupled design to further reduce computational complexity. However, this approach accumulates error from stage to stage, particularly if early-stage estimates are inaccurate.

Similar to Section 3.1.1 and with considering *U* non-overlapping sub-UPAs, the resulting AoA estimation complexity scales linearly with *U*, yielding $O\;\left(U\;{P_{{\tilde{\theta }}_{u}}}^{\left(j\right)}{P_{{\tilde{\phi }}_{u}}}^{\left(j\right)}\right)$ for the hierarchical approach, or $O\;\left(U\left(P_{{\tilde{\theta }}_{u}}+SP_{{\tilde{\phi }}_{u}}\right)\right)$ for the decoupled approach. In addition, a combining stage is required to fuse the *U* sub-UPA estimates into a final AoA estimate, which results in a complexity of O(U). Therefore, the total computational complexity is $O\;\left(U\;{P_{{\tilde{\theta }}_{u}}}^{\left(j\right)}{P_{{\tilde{\phi }}_{u}}}^{\left(j\right)}+U\right)$ for the hierarchical method, or $O\;\left(U\left(P_{{\tilde{\theta }}_{u}}+SP_{{\tilde{\phi }}_{u}}\right)+U\right)$ for the decoupled method, both of which are dominated by $O\;\left(U\;{P_{{\tilde{\theta }}_{u}}}^{\left(j\right)}{P_{{\tilde{\phi }}_{u}}}^{\left(j\right)}\right)$ and $O\;\left(U\left(P_{{\tilde{\theta }}_{u}}+SP_{{\tilde{\phi }}_{u}}\right)\right)$, respectively.

### 3.2. Self-Localization Using the NLLS Algorithm: An Infrastructure Perspective

In this subsection, we estimate the robot position at each epoch using snapshot localization, which is presented in Algorithm 1. Given that the considered scenario operates in an indoor environment at 250 GHz, the near-field scenario should be considered. To address this complexity, the AoA and RToF are estimated at the level of the *u*th sub-UPA, where the far-field approximation remains valid. Subsequently, these intermediate estimates are combined to yield final parameter estimates, which are then utilized as inputs to the localization or tracking algorithms.

Since the measurement function is nonlinear, the NLLS estimator is adopted. A linearized LS approximation can also be derived by converting (2) into a system of linear equations. However, this approximation is suboptimal, particularly under high measurement noise or poor anchor distribution.
**Algorithm 1: **3D Snapshot Self-Localization
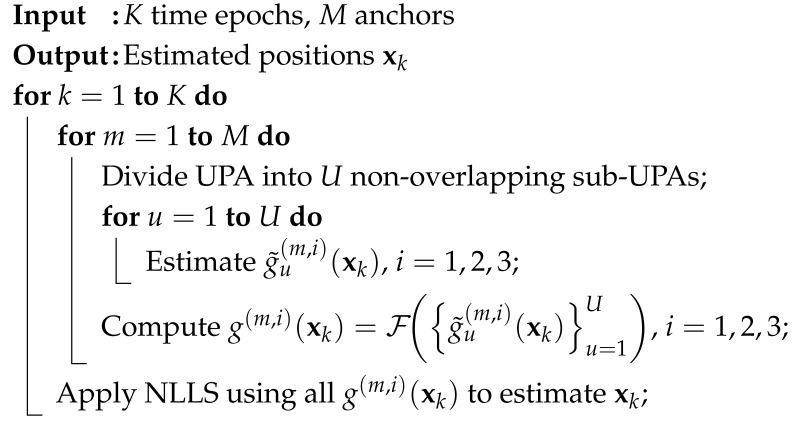


Our localization problem can be described by (2), where x is unknown but deterministic [49]. g(x) is a nonlinear vector-valued function of x, making localization a non-convex problem with many local optima. A straightforward approach is to apply NLLS estimation, as described below.

If the robot’s state $x_{k-1}$ is to be estimated, the measurement vector $z_{k-1}$ is used. When the target evolves to the next state $x_{k}$, new and independent measurements $z_{k}$ are collected to estimate $x_{k}$. Since $z_{k-1}$ and $z_{k}$ are independent, there is no need to store past measurements at epoch *k*. This scheme is called snapshot localization, which relies solely on the current measurements. Applying this approach to the mobile robot and based on (2), the robot’s state estimate at epoch *k*, ${\hat{x}}_{k}$, using the NLLS algorithm is given by [38]

(7) $$\begin{array}{cc} {\hat{x}}_{k}\; & =\arg\min_{{\hat{x}}_{k}^{-}}\left\{{∥z_{k}-g\left({\hat{x}}_{k}^{-}\right)∥}^{2}\right\}\\ \; & =\arg\min_{{\hat{x}}_{k}^{-}}\left\{\sum_{m=1}^{M}\sum_{i=1}^{I}{\left(z_{k}^{\left(m,i\right)}-g^{\left(m,i\right)}\left({\hat{x}}_{k}^{-}\right)\right)}^{2}\right\}. \end{array}$$

In general, there is no closed-form solution for (7), but $g({\hat{x}}_{k}^{-})$ can be approximated using Taylor series, and hence, the solution of (7) is written as [38]

(8) $$\begin{array}{c} {\hat{x}}_{k}={\hat{x}}_{k}^{-}+{\left(G_{k}^{T}R_{k}^{-1}G_{k}\right)}^{-1}G_{k}^{T}R_{k}^{-1}\left[z_{k}-g\left({\hat{x}}_{k}^{-}\right)\right], \end{array}$$

where ${\hat{x}}_{k}^{-}$ is a prior estimate of ${\hat{x}}_{k}$, $G_{k}\in R^{3M\times 3}$ is the Jacobian matrix of $g(x_{k})$ calculated at ${\hat{x}}_{k}^{-}$, and $R_{k}\in R^{3M\times 3M}$ is the measurement-uncertainty matrix. On the other side, the covariance matrix $P_{k}$ of the LS estimate ${\hat{x}}_{k}$ is given by [38]:

(9) $$\begin{array}{c} P_{k}={\left(G_{k}^{T}R_{k}^{-1}G_{k}\right)}^{-1}. \end{array}$$

To obtain an accurate estimate, it follows from (9) that smaller measurement noise, i.e., smaller $R_{k}$ (small/large matrix in the element-wise sense) reduces estimation uncertainty, and vice versa. Besides, a well-conditioned $G_{k}$ is required; the best case is when $G_{k}$ is full row-rank, i.e., it has linearly independent rows, which leads to smaller $P_{k}$.

Additionally, the impact of the number of anchors *M* on the estimate ${\hat{x}}_{k}$ is evident in (7) and also in (8), since the size of $z_{k}$, $G_{k}$, $g({\hat{x}}_{k}^{-})$, and $R_{k}$ is a function of *M*. Mathematically, in the proposed hybrid scheme, 3M equals the number of available equations. For a solvable problem, it is required that $3M\geq \mathrm{length}(x_{k})$. As *M* increases, more rows are added to $G_{k}$, which increases information content and reduces covariance. Roughly, the mean squared error (MSE) of the estimate ${\hat{x}}_{k}$ is inversely proportional to 3M, as will be demonstrated in Section 3.3. Consequently, increasing the number of measurements generally enhances the accuracy and robustness of the estimation against noise, provided that the measurements are independent and non-redundant.

### 3.3. Localization Performance Bounds

The Fisher information matrix (FIM) provides a theoretical lower bound for attainable localization accuracy and also offers insights into the estimation problem. Larger FIM entries generally indicate that better estimation performance can be achieved, while a more diagonal FIM indicates low correlation between the estimated parameters. In this subsection, a closed-form FIM is derived for hybrid self-localization measurements, where both RToF and AoA are exploited. In addition, we discuss the factors that affect the localization of FIM, namely anchor geometry and measurement accuracy.

#### 3.3.1. Hybrid FIM

Let $d={\left[d^{\left(1\right)},d^{\left(2\right)},...,d^{\left(M\right)}\right]}^{T}$, and $\theta ={\left[\theta^{\left(1\right)},\phi^{\left(1\right)},\theta^{\left(2\right)},\phi^{\left(2\right)},...,\theta^{\left(M\right)},\phi^{\left(M\right)}\right]}^{T}$ denote the range and AoA measurement vectors, respectively. The FIM for 3D position estimation with independent distance observations is given by [49]

(10) $$\begin{array}{cc} J_{d}\left(x\right) & ={\left(\frac{\partial d}{\partial p}\right)}^{T}{R_{d}}^{-1}\left(\frac{\partial d}{\partial p}\right). \end{array}$$

With independent AoA measurements, the FIM is written as

(11) $$\begin{array}{c} J_{\theta }\left(x\right)={\left(\frac{\partial \theta }{\partial p}\right)}^{T}{R_{\theta }}^{-1}\left(\frac{\partial \theta }{\partial p}\right), \end{array}$$

where $R_{d}=\mathrm{diag}\left[{\left(\sigma_{d}^{\left(1\right)}\right)}^{2},{\left(\sigma_{d}^{\left(2\right)}\right)}^{2},\ldots ,{\left(\sigma_{d}^{\left(M\right)}\right)}^{2}\right]$, and $R_{\theta }=\mathrm{diag}{\left[{\left(\sigma_{\theta }^{\left(1\right)}\right)}^{2},{\left(\sigma_{\phi }^{\left(1\right)}\right)}^{2},(\sigma_{\theta }^{\left(2\right)}\right)}^{2},{\left(\sigma_{\phi }^{\left(2\right)}\right)}^{2},\ldots ,{\left(\sigma_{\theta }^{\left(M\right)}\right)}^{2},{\left(\sigma_{\phi }^{\left(M\right)}\right)}^{2}]$. However, the measurement noise variances are lower bounded by the Cramér–Rao lower bound (CRLB), which is

(12) $$\begin{array}{c} {\left(\sigma_{d}^{\left(m\right)}\right)}^{2}\geq \mathrm{CRLB}\left(d^{\left(m\right)}\right)≜\frac{c^{2}}{4B^{2}N\;{\mathrm{SNR}}^{\left(m\right)}}, \end{array}$$

and

(13) $$\begin{array}{c} \left[\begin{array}{c} {\left(\sigma_{\theta }^{\left(m\right)}\right)}^{2}\\ {\left(\sigma_{\phi }^{\left(m\right)}\right)}^{2} \end{array}\right]\geq \mathrm{CRLB}\left(\theta^{\left(m\right)}\right)≜\frac{6}{\pi^{2}N\left(N-1\right){\mathrm{SNR}}^{\left(m\right)}{\left[\cos^{2}\phi_{k}^{\left(m\right)},\sin^{2}\phi_{k}^{\left(m\right)}\right]}^{T}}, \end{array}$$

where *B* is the operating bandwidth in Hz, and ${\mathrm{SNR}}^{\left(m\right)}$ is the received signal-to-noise ratio for the $m^{th}$ anchor. Note that (13) is an approximated formula for a square UPA where $N_{a}=N_{b}=\sqrt{N}$ [50]. Increasing *N* improves both time and angle estimation, with a stronger effect on angle estimation. For example, doubling the number of antennas from *N* to 2N improves the AoA estimation bound by a factor of two. Simplified closed forms for $J_{d}\left(x\right)$ and $J_{\theta }\left(x\right)$ are shown in Appendix A and Appendix B, respectively.

Based on (10) and (11), the hybrid FIM is

(14) $$\begin{array}{ccc} J_{h}\left(p\right) & = & J_{d}\left(p\right)+J_{\theta }\left(p\right). \end{array}$$

It is clear that $J_{h}\left(p\right)⪰J_{d}\left(p\right)$ and $J_{h}\left(p\right)⪰J_{\theta }\left(p\right)$. Consequently, hybrid observations enhance the FIM by providing additional information, thereby supporting the objective of accurate localization with minimal infrastructure. Mathematically, even if one component FIM is singular, the hybrid FIM may still be nonsingular if the two measurement types provide complementary information.

To quantify how the CRLB improves with the hybrid scheme, we apply the matrix inversion lemma to (14). The inverse is

(15) $$\begin{array}{cc} J_{h}^{-1}\left(p\right)= & J_{d}^{-1}\left(p\right)\;-[J_{d}^{-1}\left(p\right){\left(\frac{\partial \theta }{\partial p}\right)}^{T}\times \\ \; & {\left(R_{\theta }+\left(\frac{\partial \theta }{\partial p}\right)J_{d}^{-1}\left(p\right){\left(\frac{\partial \theta }{\partial p}\right)}^{T}\right)}^{-1}\left(\frac{\partial \theta }{\partial p}\right)J_{d}^{-1}\left(p\right)]. \end{array}$$

Since ${\mathrm{CRLB}}_{h}\left(p\right)=\mathrm{tr}\left[J_{h}^{-1}\left(p\right)\right]$, (15) shows that the hybrid bound is no worse than the range-only bound under the stated assumptions. This reduction depends on $R_{\theta }$ and the geometry matrix $\frac{\partial \theta }{\partial p}$, which will be discussed next in Section 3.3.2.

As $R_{\theta }\to 0$ with nonsingular $\frac{\partial \theta }{\partial p}$, ${\mathrm{CRLB}}_{h}\to 0$ in (15). Similarly, when $R_{\theta }\to \infty$, ${\mathrm{CRLB}}_{h}\to {\mathrm{CRLB}}_{d}$.

**Proposition** **1.** *Let X≻0 and Y≻0; then*

(16) $$\begin{array}{c} \mathrm{tr}\left[{\left(X+Y\right)}^{-1}\right]<\min\left(\mathrm{tr}\left[X^{-1}\right],\mathrm{tr}\left[Y^{-1}\right]\right). \end{array}$$

The proof is given in Appendix C.

#### 3.3.2. Impact of Anchor Geometry

The Jacobian matrix $G^{0}$ under the hybrid scheme can be written as

(17) $$\begin{array}{cc} G^{0}= & {\left[\frac{\partial d}{\partial p},\frac{\partial \theta }{\partial p}\right]}^{T}. \end{array}$$

Here, $G^{0}$ is the geometry matrix evaluated at x, whereas G in Section 3.2 is the same matrix but evaluated at $\hat{x}$. Moreover, the covariance matrix of the measurements is $R_{h}=\mathrm{blkdiag}\left[R_{d},R_{\theta }\right]$.

However, $J_{h}\left(p\right)$ depends on both $R_{h}$ and $G^{0}$. The geometry matrix $G^{0}$ is determined by the spatial distribution of anchors around the target. To focus on the impact of anchor geometry, we assume that the anchors are distributed such that the measurement uncertainty is the same for all anchors, i.e., ${\sigma_{\nu }}^{\left(b\right)}=\sigma_{\nu }$ for b=1,2,...,3M, ν={d,θ,ϕ}. Hence $R_{h}={\sigma_{\nu }}^{2}I_{3M}$. Accordingly, $J_{h}\left(p\right)$ is simplified to

(18) $$\begin{array}{c} J_{h}\left(p\right)=\frac{3M}{{\sigma_{\nu }}^{2}}{G^{0}}^{T}G^{0}. \end{array}$$

An important metric for quantifying the effect of anchor geometry on position accuracy is geometric dilution of precision (GDOP). GDOP describes how measurement errors are amplified into position estimation errors depending on anchor geometry. It is given by [38]

(19) $$\begin{array}{c} \mathrm{GDOP}=\sqrt{\mathrm{tr}\left[{\left({G^{0}}^{T}G^{0}\right)}^{-1}\right]}=\frac{\mathrm{PEB}}{\sigma_{s}}, \end{array}$$

where the position error bound (PEB) is given by (the PEB is expressed in meters and is defined as $\mathrm{PEB}=\sqrt{\mathrm{CRLB}(p)}≜\sqrt{\mathrm{tr}\left[J^{-1}\left(p\right)\right]}$)

$$\begin{array}{c} \mathrm{PEB}=\frac{\sigma_{\nu }}{\sqrt{3M}}\sqrt{\mathrm{tr}\left[{(G^{0}}^{T}G^{0}{)}^{-1}\right]}, \end{array}$$

and

$$\begin{array}{c} \sigma_{s}=\sqrt{\frac{1}{3M}\sum_{b=1}^{3M}{\left(\sigma_{\nu }^{\left(b\right)}\right)}^{2}}=\frac{\sigma_{\nu }}{\sqrt{3M}},\mathrm{when}\;\sigma_{\nu }^{\left(b\right)}=\sigma_{\nu }. \end{array}$$

The value of GDOP is dimensionless and depends solely on geometry when measurement noise is equal. Poor anchor geometry (e.g., anchors aligned in a line) makes $G^{0}$ nearly singular, yielding a large GDOP and poor accuracy despite low noise. Favorable geometry is obtained when anchors are well distributed around the target, which improves the conditioning of $G^{0}$, ensures full rank, and reduces the position error. To improve GDOP and enrich the spatial geometry of the anchors, we impose another constraint on the placement optimization algorithm described in [14], which is to select the anchors from different planes.

To generalize (19) to the case of unequal noise levels, GDOP is expressed as

(20) $$\begin{array}{c} \mathrm{GDOP}=\frac{\sqrt{\mathrm{tr}\left[{\left({G^{0}}^{T}{R_{h}}^{-1}G^{0}\right)}^{-1}\right]}}{\sigma_{s}}. \end{array}$$

Now, GDOP reflects both geometry and measurement quality: anchors with higher measurement accuracy contribute more information. Increasing the number of anchors reduces GDOP, provided geometry is favorable and measurement noise is low. However, beyond a certain number, the improvement diminishes. In practice, GDOP is calculated using G rather than $G^{0}$, since the true location p is unknown (hence, the GDOP is given by $\mathrm{GDOP}=\frac{\sigma_{p}}{\sigma_{s}}$, where $\sigma_{p}$ is the RMSE of the position estimation).

#### 3.3.3. Impact of the Number and Accuracy of Measurements

Let the localization root mean squared error (RMSE) be denoted by $\sigma_{p}$. In order to achieve the desired value of $\sigma_{p}$, it is important to know the required value of the measurement RMSE. To simplify the analysis, we assume excellent anchor geometry (minimum GDOP) and identical range measurement uncertainty $\sigma_{d}^{\left(m\right)}=\sigma_{d}$ for all anchors. Then

(21) $$\begin{array}{c} \sigma_{p-ToF}\approx \frac{\sigma_{d}}{\sqrt{M}}. \end{array}$$

Thus, $\sigma_{p}$ is linearly proportional to $\sigma_{d}$, and inversely proportional to $\sqrt{M}$.

Similarly to (21), the dependency of $\sigma_{p}$ on the AoA RMSE $\sigma_{\theta }$ under the assumption that $\sigma_{\theta }=\sigma_{\theta }^{\left(m\right)}=\sigma_{\phi }^{\left(m\right)}$ using AoA measurements is given by

(22) $$\begin{array}{c} \sigma_{p-AoA}\approx \frac{d\sigma_{\theta }}{\sqrt{2M}}, \end{array}$$

where *d* is the distance in (m) between the robot and the anchors. Again, the impact of $\sigma_{\theta }$ is shown in (22), and in principle, $\sigma_{\theta }$ should be sufficiently small relative to $\sigma_{d}$ to achieve the same performance as in (21), while the effect of *M* is doubled.

In the hybrid case, $\sigma_{p}$ is given by (under the same assumption as in (21) and (22))

(23) $$\begin{array}{ccc} \sigma_{p-h} & \approx & \sqrt{{\left(\frac{1}{\sigma_{p-ToF}^{2}}+\frac{1}{\sigma_{p-AoA}^{2}}\right)}^{-1}}. \end{array}$$

Accordingly, if $\sigma_{d}<<d\sigma_{\theta }$, then $\sigma_{p-h}\approx \frac{\sigma_{d}}{\sqrt{M}}$, while if $d\sigma_{\theta }<<\sigma_{d}$, then $\sigma_{p-h}\approx \frac{d\sigma_{\theta }}{\sqrt{2M}}$, and finally if $\sigma_{d}\approx d\sigma_{\theta }$, then $\sigma_{p-h}\approx \frac{\sigma_{d}}{\sqrt{3M}}$. Under these assumptions, the hybrid scheme exhibits an RMSE approximately proportional to $\sigma_{p}\propto \frac{1}{\sqrt{3M}}$.

## 4. Tracking Algorithm with Reduced Infrastructure Dependency

This section develops the EKF-based tracking formulation and analyzes how the number of available measurements affects tracking performance and the corresponding theoretical bounds. This relationship is used to study the infrastructure required to achieve a desired accuracy level. This section also compares dynamic tracking with snapshot localization.

### 4.1. Robot Tracking Using the Extended Kalman Filter

The EKF is based on first-order linearization of the nonlinear state-transition and measurement models around the current estimate, followed by standard Kalman filter updates [5]. Similar to Section 3.2, the measurement vector $z_{k}$ is obtained by combining the measurements from all sub-UPAs.

At each epoch, the filter first predicts the next state using the process model and then corrects that prediction using the new measurements. Accordingly, the error covariance typically increases during prediction and decreases after the measurement update.

The EKF recursion used in this work is summarized in Algorithm 2. In Algorithm 2, $F_{k-1}$ is the Jacobian matrix of f(.) evaluated at ${\hat{x}}_{k-1}$ and $u_{k}$. $G_{k}$ is the Jacobian of g(.) evaluated at the predicted state ${\hat{x}}_{k}^{-}$. The matrix $K_{k}$ is the Kalman filter gain at epoch *k*.
**Algorithm 2: **Extended Kalman Filter (EKF) Algorithm**Input**: Previous state ${\hat{x}}_{k-1}$, previous covariance $P_{k-1}$, control input $u_{k}$, measurement $z_{k}$**Output**: Updated state estimate ${\hat{x}}_{k}$, updated covariance $P_{k}$${\hat{x}}_{k}^{-}\leftarrow f\left(u_{k},{\hat{x}}_{k-1}\right);$$P_{k}^{-}\leftarrow F_{k-1}P_{k-1}F_{k-1}^{T}+Q_{k-1};$$K_{k}\leftarrow P_{k}^{-}G_{k}^{T}{\left(G_{k}P_{k}^{-}G_{k}^{T}+R_{k}\right)}^{-1};$${\hat{x}}_{k}\leftarrow {\hat{x}}_{k}^{-}+K_{k}\left(z_{k}-g\left({\hat{x}}_{k}^{-}\right)\right);$$P_{k}\leftarrow \left(I-K_{k}G_{k}\right)P_{k}^{-};$**return** ${\hat{x}}_{k},P_{k};$**Repeat** for k+1;

A larger measurement covariance $R_{k}$ generally reduces the influence of the measurements, whereas larger prediction uncertainty, reflected by $P_{k}^{-}$ and $Q_{k}$, generally increases it. The Kalman gain therefore governs the balance between model-based prediction and measurement-based correction.

### 4.2. Extended Kalman Filter Performance from an Infrastructure Perspective

In general, increasing the number of informative measurements enlarges the measurement vector and can improve the conditioning of the measurement update. This typically reduces the posterior covariance $P_{k}$ and can accelerate convergence, provided that the added measurements are sufficiently accurate and non-redundant.

At the beginning of the recursion, the filter often starts with relatively high uncertainty $P_{0}$, which produces a large $K_{k}$ and makes the early updates more sensitive to the measurements. As more measurements are processed, the estimated uncertainty $P_{k}$ decreases, $K_{k}$ is reduced, and the filter relies more on the mobility model, requiring fewer measurements.

For a more detailed analysis of how the number of measurements affects KF performance, we consider two simple scenarios: a 1D EKF case and a 2D linear KF case.

- 1D Scenario: Assume that a 1D position is to be estimated, i.e., $\mathrm{length}(x_{k})=1$. The number of observations increases from one to three. This illustrates how the Kalman gain changes as the number of measurements increases. At k=1, the initial estimate is $x_{0}$ with uncertainty $P_{0}={\sigma_{0}}^{2}$, ${\hat{x}}_{1}^{-}=x_{0}$, $F_{k-1}=1$, and $Q_{k}={\sigma_{x}}^{2}$, giving $P_{1}^{-}={\sigma_{0}}^{2}+{\sigma_{x}}^{2}$, where ${\sigma_{0}}^{2}\neq 0$ and ${\sigma_{x}}^{2}\neq 0$.
  - Case 1: One observation (β=M=1):
    (24) $$\begin{array}{ccc} R_{k} & = & {\sigma_{\nu }}^{2},{\sigma_{\nu }}^{2}\neq 0\\ K_{1} & = & \frac{{\sigma_{0}}^{2}+{\sigma_{x}}^{2}}{{\sigma_{0}}^{2}+{\sigma_{x}}^{2}+{\sigma_{\nu }}^{2}},\\ P_{1} & = & \frac{\left({\sigma_{0}}^{2}+{\sigma_{x}}^{2}\right){\sigma_{\nu }}^{2}}{{\sigma_{0}}^{2}+{\sigma_{x}}^{2}+{\sigma_{\nu }}^{2}}. \end{array}$$
  - Case 2: Two observations (β=2). Similar to Case 1:
    (25) $$\begin{array}{ccc} K_{1} & = & \frac{{\sigma_{0}}^{2}+{\sigma_{x}}^{2}}{2{\sigma_{0}}^{2}+2{\sigma_{x}}^{2}+{\sigma_{\nu }}^{2}}\left[\begin{array}{cc} 1 & 1 \end{array}\right],\\ P_{1} & = & \frac{\left({\sigma_{0}}^{2}+{\sigma_{x}}^{2}\right){\sigma_{\nu }}^{2}}{2{\sigma_{0}}^{2}+2{\sigma_{x}}^{2}+{\sigma_{\nu }}^{2}}. \end{array}$$
  - Case 3: Three observations (β=3). Following the same steps as in Cases 1 and 2:
    (26) $$\begin{array}{ccc} K_{1} & = & \frac{{\sigma_{0}}^{2}+{\sigma_{x}}^{2}}{3{\sigma_{0}}^{2}+3{\sigma_{x}}^{2}+{\sigma_{\nu }}^{2}}\left[\begin{array}{ccc} 1 & 1 & 1 \end{array}\right],\\ P_{1} & = & \frac{\left({\sigma_{0}}^{2}+{\sigma_{x}}^{2}\right){\sigma_{\nu }}^{2}}{3{\sigma_{0}}^{2}+3{\sigma_{x}}^{2}+{\sigma_{\nu }}^{2}}. \end{array}$$
- 2D Scenario: Now consider 2D position estimation. Again, the number of observations increases from one to three. At k=1, the initial estimate is $x_{0}={\left[\begin{array}{cc} x_{0}, & y_{0} \end{array}\right]}^{T}$ with uncertainty $P_{0}={\sigma_{0}}^{2}I_{2}$, ${\hat{x}}_{1}^{-}=x_{0}$, $F_{k-1}=I_{2}$, and
  $$\begin{array}{c} Q_{k}=\left[\begin{array}{cc} {\sigma_{x}}^{2} & 0\\ 0 & {\sigma_{y}}^{2} \end{array}\right]={\sigma_{x}}^{2}I_{2},\;{\sigma_{x}}^{2}={\sigma_{y}}^{2}, \end{array}$$
  giving $P_{1}^{-}=\left({\sigma_{0}}^{2}+{\sigma_{x}}^{2}\right)I_{2}$, where ${\sigma_{0}}^{2}\neq 0$ and ${\sigma_{x}}^{2}\neq 0$. Then, similarly to the previous 1D scenario, we obtain:
  - Case 1: One observation (β=M=1):
    (27) $$\begin{array}{ccc} K_{1} & = & \frac{{\sigma_{0}}^{2}+{\sigma_{x}}^{2}}{2{\sigma_{0}}^{2}+2{\sigma_{x}}^{2}+{\sigma_{\nu }}^{2}}\left[\begin{array}{c} 1\\ 1 \end{array}\right],\\ P_{1} & = & \frac{\left({\sigma_{0}}^{2}+{\sigma_{x}}^{2}\right)\left({\sigma_{0}}^{2}+{\sigma_{x}}^{2}+{\sigma_{\nu }}^{2}\right)}{2{\sigma_{0}}^{2}+2{\sigma_{x}}^{2}+{\sigma_{\nu }}^{2}}I_{2}. \end{array}$$
  - Case 2: Two observations (β=2):
    (28) $$\begin{array}{ccc} K_{1} & = & \frac{{\sigma_{0}}^{2}+{\sigma_{x}}^{2}}{4{\sigma_{0}}^{2}+4{\sigma_{x}}^{2}+{\sigma_{\nu }}^{2}}\left[\begin{array}{cc} 1 & 1\\ 1 & 1 \end{array}\right],\\ P_{1} & = & \frac{\left({\sigma_{0}}^{2}+{\sigma_{x}}^{2}\right)\left(2{\sigma_{0}}^{2}+2{\sigma_{x}}^{2}+{\sigma_{\nu }}^{2}\right)}{4{\sigma_{0}}^{2}+4{\sigma_{x}}^{2}+{\sigma_{\nu }}^{2}}I_{2}. \end{array}$$
  - Case 3: Three observations (β=3):
    (29) $$\begin{array}{ccc} K_{1} & = & \frac{{\sigma_{0}}^{2}+{\sigma_{x}}^{2}}{6{\sigma_{0}}^{2}+6{\sigma_{x}}^{2}+{\sigma_{\nu }}^{2}}\left[\begin{array}{ccc} 1 & 1 & 1\\ 1 & 1 & 1 \end{array}\right],\\ P_{1} & = & \frac{\left({\sigma_{0}}^{2}+{\sigma_{x}}^{2}\right)\left(3{\sigma_{0}}^{2}+3{\sigma_{x}}^{2}+{\sigma_{\nu }}^{2}\right)}{6{\sigma_{0}}^{2}+6{\sigma_{x}}^{2}+{\sigma_{\nu }}^{2}}I_{2}. \end{array}$$

From (24)–(29), note that $K_{k}$ is scalar in Case 1, whereas it becomes a row vector in Case 2 and a matrix in Case 3. This implies that as more measurements are included, their contribution to the final estimate increases, while $P_{k}$ decreases, improving accuracy. Assuming that the measurement residuals $\left(z_{k}-g\left({\hat{x}}_{k}^{-}\right)\right)$ are identical for all measurements, the denominator of $K_{k}$ decreases with a factor determined by $\frac{{\sigma_{\nu }}^{2}}{M}$. Hence, the fraction of measurement residuals contributing to the estimate increases as *M* grows. For example in the 1D scenario, for very small *M*, the measurement contribution $\frac{{\sigma_{0}}^{2}+{\sigma_{x}}^{2}}{{\sigma_{0}}^{2}+{\sigma_{x}}^{2}+\frac{{\sigma_{\nu }}^{2}}{M}}\to 0$. Conversely, for very large *M*, $\frac{{\sigma_{0}}^{2}+{\sigma_{x}}^{2}}{{\sigma_{0}}^{2}+{\sigma_{x}}^{2}+\frac{{\sigma_{\nu }}^{2}}{M}}\to 1$.

### 4.3. Tracking Performance Bounds

The Bayesian filtering performance bound depends on both the measurements and the mobility model, and is generally tighter than that of snapshot localization, since it exploits the motion model. This becomes evident by comparing the expression for $P_{k}$ in Algorithm 2 with (9).

For simplicity, we write $J_{k}$ instead of $J(x_{k})$, where [51]

(30) $$\begin{array}{c} J_{k}=\underset{\mathrm{Prior}\mathrm{FIM}}{\underset{︸}{{\left(F_{k-1}^{0}J_{k-1}^{-1}{F_{k-1}^{0}}^{T}+Q_{k-1}\right)}^{-1}}}+\underset{\mathrm{Measurements}\mathrm{FIM}}{\underset{︸}{{G_{k}^{0}}^{T}R_{k}^{-1}G_{k}^{0}}}. \end{array}$$

It can be shown that (30) is identical to the $P_{k}$ expression in Algorithm 2, with $P_{k}$ replaced by $J_{k}^{-1}$, and the Jacobians $F_{k-1}^{0}$ and $G_{k}^{0}$ evaluated at the true values. Additional details are provided in [52].

The impact of the number of measurements on the Bayesian FIM is evident from (30). As the number of measurements increases, the second term in (30) becomes larger, which leads to a larger FIM and a lower CRLB.

For comparison, the FIM for the static case is given as in Section 3.3

(31) $$\begin{array}{c} H_{k}={G_{k}^{0}}^{T}R_{k}^{-1}G_{k}^{0}. \end{array}$$

As $Q_{k-1}\to \infty$, the FIM of the dynamic case in (30) reduces to the static case as in (31) [40], since the process noise compensates for uncertainty in the mobility model. This implies that the mobility model provides no information and is treated as entirely random. In this case, the estimator relies solely on the measurements.

In contrast, when $R_{k}\to 0$, the measurement term in (30) becomes dominant. However, when $R_{k}\to \infty$, this term vanishes. Increasing the number of measurements provides more information, since the information is additive [40], which improves estimation accuracy and reduces the CRLB.

In dynamic scenarios, however, the mobility model serves as an additional source of information, reducing the dependence on measurements compared to static cases. This makes it possible to track an object even when the number of observations is smaller than the number of unknowns. A well-designed KF allows effective control of the filter behavior, enabling one to prioritize the measurements or the motion model at any given moment.

## 5. Simulation Results

This section evaluates the proposed THz chipless RFID-based self-localization and tracking system through simulation. The evaluation focuses on the proposed RToF-AoA scheme and examines how localization and tracking accuracy depend on infrastructure density, measurement quality, and anchor geometry.

A 3D indoor simulation environment consistent with Figure 1 is considered in the simulations. The robot is equipped with a UPA that is partitioned into non-overlapping square sub-UPAs, each having a fixed size of 5×5. The reported parameters θ, ϕ, and *d* correspond to the final estimates obtained by applying simple averaging to the individual estimates from all sub-UPAs. The robot moves along the nonlinear trajectory defined in (3), with initial position $p_{0}={\left[2.33,2.77,2.57\right]}^{T}$ m and $P_{0}=\mathrm{diag}\left[0.1^{2},0.1^{2},0.1^{2}\right]$. The initial orientations are ξ=π/6 rad and ψ=0 rad. The main simulation parameters are listed in Table 1. All measurements are assumed to be mutually independent, and the process-noise standard deviations in the x, y, and z directions are identical, i.e., ${\sigma_{q}}_{x}={\sigma_{q}}_{y}={\sigma_{q}}_{z}=\sigma_{q}$. The anchor positions are assumed to be fixed, adopted from [14] and perfectly known throughout the simulation environment.

Based on (21), and under the minimum anchor count *M*, sub-mm localization accuracy in the present geometry requires approximately $\sigma_{d}<1\;\mathrm{mm}$. Similarly, from (22), maintaining the same level of accuracy demands AoA measurement noise below $\sigma_{\theta }<10^{-4}\;\mathrm{rad}\;\left(\approx 0.006^{\circ }\right)$ in both azimuth and elevation, considering a maximum distance of $d_{\max}=14.46\;m$, and the average distance $\bar{d}$ is approximately 7m in our setup.

### 5.1. Localization Results

This subsection examines the dependency of localization accuracy on the available infrastructure in a stationary robot scenario, in which the robot remains fixed at an unknown position. The three localization schemes—RToF, AoA, and the hybrid scheme—are compared in terms of achievable 3D localization accuracy under the minimum anchor configurations considered in this work. RToF requires at least four anchors in 3D, AoA-based localization requires at least two anchors, whereas the hybrid scheme can, under the adopted assumptions, estimate the 3D position with a single anchor.

**Figure 3:**
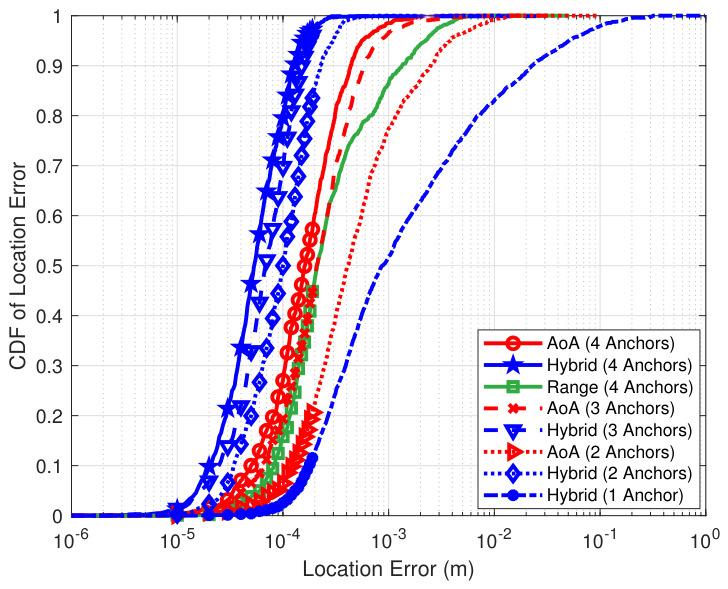
The CDF of the 3D position error for different localization schemes.

Figure 3 depicts the cumulative distribution function (CDF) of the localization error for the three measurement schemes. For the hybrid scheme, the probability that the localization error is below $8\times 10^{-4}\;m$ is 50% for one anchor and reaches 100% for two or more anchors. For the RToF scheme, at least four anchors are needed to achieve an error below $8\times 10^{-4}\;m$ with 83% confidence. Using the AoA scheme, the same error threshold is achieved with 95% confidence for three anchors and 97.5% for four anchors. These results highlight the trade-off between localization accuracy and the number of anchors, as more anchors yield additional independent measurements and consequently more information for estimation. Nevertheless, the proposed hybrid scheme outperforms the other schemes, as it achieves high localization accuracy with significantly lower infrastructure dependence compared to the RToF and AoA schemes.

**Figure 4:**
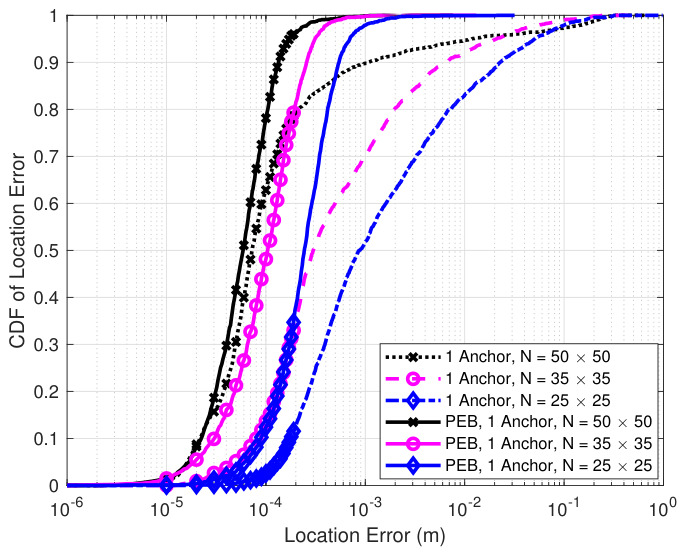
The CDF of the 3D position error and the corresponding PEB for hybrid scheme, with single anchor and different numbers of antennas *N*.

Figure 4 presents the CDF of the localization error and the corresponding PEB for the hybrid scheme using a single anchor for different numbers of antenna elements *N*. When N=25×25, the location error is below $8\times 10^{-4}\;m$ at 50% confidence, and the probability that the $\mathrm{PEB}\leq 8\times 10^{-4}\;m$ is 96.5%. Increasing *N* to 35×35 raises the confidence to 68%, and the probability that the PEB remains below this threshold increases to 99.6%. Finally, for N=50×50, the confidence that the localization error is below $8\times 10^{-4}\;m$ increases to 90%, and the PEB consistently remains below this threshold.

### 5.2. Tracking Results

Unlike the previous subsection, which considered stationary localization, this subsection evaluates dynamic tracking performance of the proposed system, where the robot moves along a predefined nonlinear trajectory. The objective is to study the influence of process noise and measurement accuracy on EKF-based tracking with limited infrastructure. Initially, the robot starts its trajectory from an initial state $x_{0}$ with a corresponding ${\mathrm{RMSE}}_{0}$.

**Figure 5:**
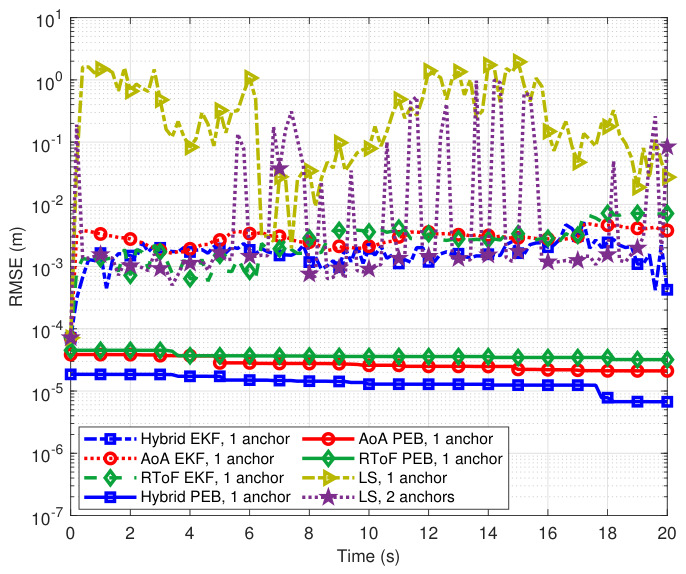
Performance comparison in terms of RMSE and PEB between EKF-based tracking using different measurement schemes and the LS estimator. The x-axis represents motion time in seconds.

Figure 5 illustrates the RMSE versus time for the robot location estimated by the EKF under different measurement schemes, along with the corresponding PEBs obtained using a single anchor. For comparison, the figure also includes the RMSE of the LS algorithm using one and two anchors. In this setup, the process noise standard deviation is $\sigma_{q}=5\;\mathrm{cm}$, and the observation noise standard deviations are $\sigma_{d}=8\times 10^{-4}\;m$, and $\sigma_{\theta }=\sigma_{\phi }=7\times 10^{-4}\;\mathrm{rad}$ for range and AoA in both azimuth and elevation, respectively. Figure 5 shows that the EKF with the hybrid scheme outperforms the RToF and AoA methods as well as the LS estimator, achieving faster convergence, greater stability, lower infrastructure dependency, and higher accuracy. Furthermore, Figure 5 shows that the PEB of the hybrid scheme is the smallest, as it leverages both range and angular measurements, thereby exploiting more information than the other two approaches. The EKF-based RMSE curves are also smoother compared to those of the LS method, which depends solely on instantaneous measurements.

**Figure 6:**
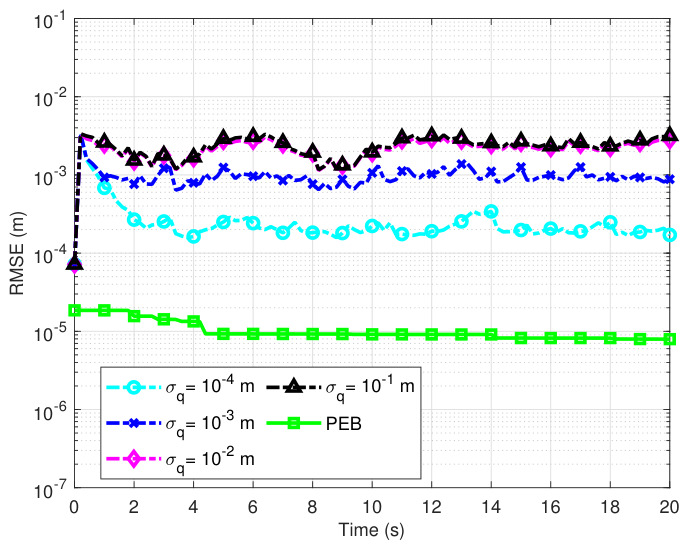
EKF tracking performance using one anchor and hybrid measurements under different values of process-noise standard deviation $\sigma_{q}$ and fixed measurement uncertainties, where $\sigma_{d}=10^{-3}\;m$ and $\sigma_{\theta }=\sigma_{\phi }=10^{-3}\;\mathrm{rad}$. The x-axis represents motion time in seconds.

Figure 6 analyzes the influence of the process-model uncertainty ($\sigma_{q}$) on the EKF RMSE when a single anchor and the hybrid measurement scheme are used. Three measurements are available per epoch with measurement uncertainties $\sigma_{d}=10^{-3}\;m$ and $\sigma_{\theta }=\sigma_{\phi }=10^{-3}\;\mathrm{rad}$. The results show that reducing the process noise substantially improves performance. For example, at $\sigma_{q}=1\;\mathrm{cm}$ or 10cm, the EKF RMSE remains within $4\times 10^{-3}$, whereas achieving sub-mm RMSE requires $\sigma_{q}\leq 1\;\mathrm{mm}$.

**Figure 7:**
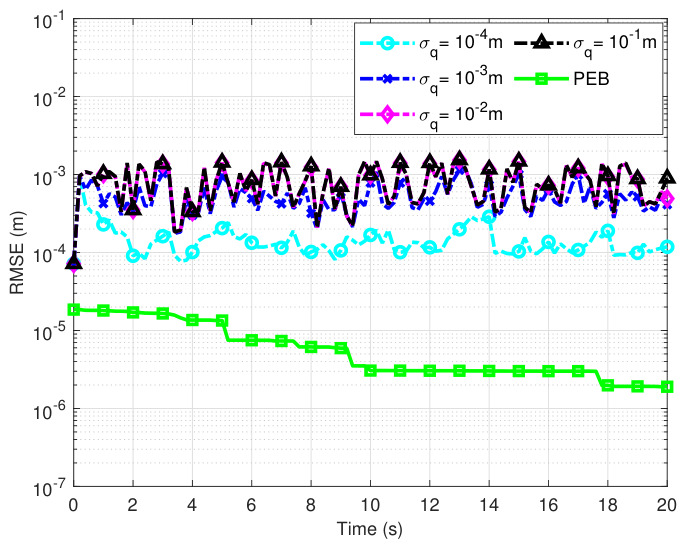
EKF tracking performance using one anchor and hybrid measurements under different values of process-noise standard deviation $\sigma_{q}$ and fixed measurement uncertainties, where $\sigma_{d}=10^{-3}\;m$ and $\sigma_{\theta }=\sigma_{\phi }=10^{-4}\;\mathrm{rad}$. The x-axis represents motion time in seconds.

**Figure 8:**
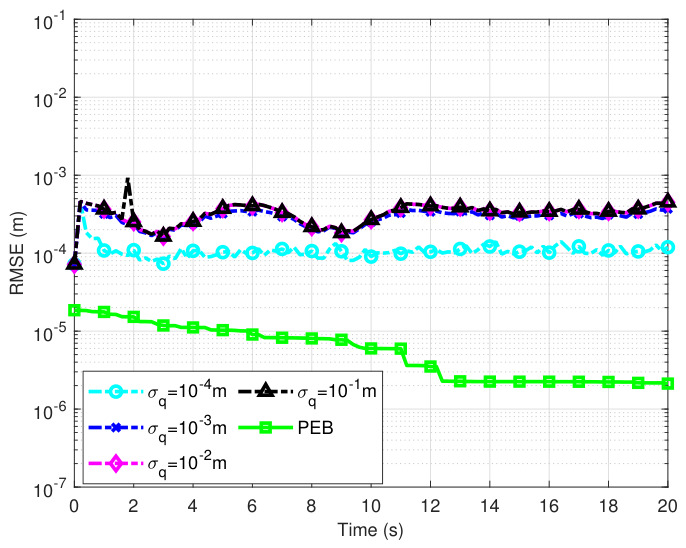
EKF tracking performance using one anchor and hybrid measurements under different values of process-noise standard deviation $\sigma_{q}$ and fixed measurement uncertainties, where $\sigma_{d}=10^{-4}\;m$ and $\sigma_{\theta }=\sigma_{\phi }=10^{-4}\;\mathrm{rad}$. The x-axis represents motion time in seconds.

Figure 7 and Figure 8 further illustrate the EKF performance in terms of RMSE and PEB under conditions of higher measurement accuracy in comparison with Figure 6. As anticipated, both RMSE and PEB decrease as the measurement precision improves, demonstrating that sub-mm localization accuracy becomes attainable even when $\sigma_{q}$ remains at the centimeter range. Another observation is that the influence of process-model uncertainty ($\sigma_{q}$) diminishes as the measurement accuracy increases. In this regime, the more accurate observations dominate the estimation performance, and only very small values of $\sigma_{q}$ have a noticeable effect.

It is also observed that the EKF performance in Figure 7 is less smooth than in Figure 6 and Figure 8, when the measurement uncertainties are $\sigma_{d}=10^{-3}\;m$ and $\sigma_{\theta }=\sigma_{\phi }=10^{-4}\;\mathrm{rad}$. Based on (23) and since $\sigma_{d}\approx \bar{d}\sigma_{\theta }$, the three observation uncertainties become comparable in magnitude and jointly dominate the estimation performance. As a result, the EKF relies almost entirely on the measurements rather than the mobility model. In this case, the tracking problem approaches snapshot localization, especially at higher $\sigma_{q}$.

Note that the tracking PEB presented in Figure 5, Figure 6, Figure 7 and Figure 8 is computed under the assumption of zero process noise. Among the considered schemes, the hybrid approach consistently yields the lowest PEB, because its Bayesian FIM is the sum of the two individual FIMs as defined in (14).

Moreover, Figure 5, Figure 6, Figure 7 and Figure 8 indicate that the Bayesian FIM either remains constant or increases with time, which can be understood from (30). Fundamentally, the Bayesian FIM is non-decreasing as the process evolves from epoch *k* to k+1, since it is the sum of two positive semi-definite matrices. When the contribution of new measurements is significant, the Bayesian FIM increases accordingly. Conversely, if the measurements provide limited information, the increase in the Bayesian FIM is negligible, and it effectively remains constant. As measurement accuracy improves, the corresponding reduction in the PEB becomes more pronounced. In contrast, inaccurate measurements exert minimal influence on the tracking PEB, which is dominated by the prior. This explains why the PEB reduction in Figure 7 and Figure 8 is more significant than in Figure 5 and Figure 6.

### 5.3. Impact of Anchor Geometry and Number on the PEB

**Figure 9:**
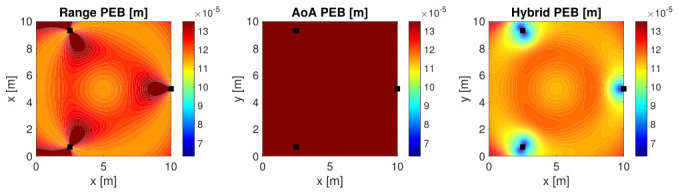
PEB distribution for the three measurement schemes (RToF, AoA, and hybrid) under a favorable anchor configuration. The anchors are shown as black squares.

**Figure 10:**
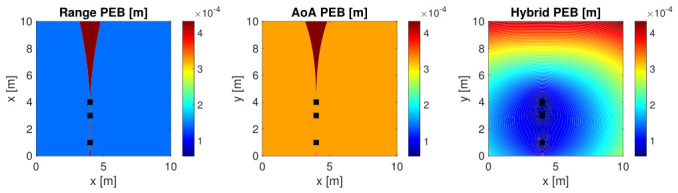
PEB distribution for the three measurement schemes (RToF, AoA, and hybrid) under a linear anchor configuration. The anchors are shown as black squares.

**Figure 11:**
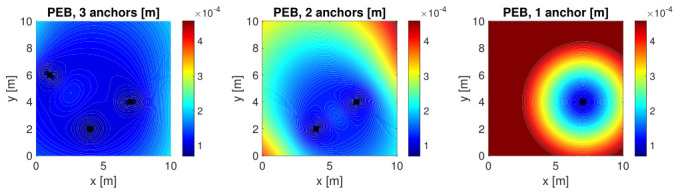
PEB distribution for hybrid measurements using three, two, and one anchor(s). The anchors are shown as black squares.

To investigate the influence of anchor placement on localization accuracy, Figure 9, Figure 10 and Figure 11 show heat maps of the PEB as a function of the robot’s 2D location under different anchor geometries. Unless otherwise stated, the measurement uncertainties are $\sigma_{d}=10^{-4}\;m$ and $\sigma_{\theta }=10^{-4}\;\mathrm{rad}$.

Figure 9 illustrates the PEB distribution for the three measurement schemes assuming a favorable anchor geometry, where anchors are symmetrically placed around the target (either in a circular configuration or at the vertices of an equilateral triangle) with the robot located at the center. This yields a small value of GDOP and, hence, a small PEB. As shown, the hybrid measurement scheme consistently achieves the lowest PEB, outperforming both individual schemes. For the RToF scheme, ambiguity appears in regions highlighted in dark red, corresponding to the highest PEB values, whereas the same regions exhibit much lower PEB under the hybrid scheme, demonstrating that combining range and angle information effectively resolves positional ambiguity.

Figure 10 shows the worst-case anchor geometry, where all anchors are linearly aligned. Even in this degenerate configuration, the hybrid approach still yields the smallest PEB and maintains a nonsingular FIM over the considered target locations. Conversely, the AoA and RToF schemes exhibit singularities (dark-red areas), indicating unidentifiable positions and poor estimation robustness. This figure further confirms that the hybrid scheme can eliminate localization ambiguity that arises when relying on a single measurement type.

Finally, Figure 11 shows the impact of the number of anchors on the PEB of the hybrid scheme for three, two, and one anchor configurations. As expected, increasing the number of anchors reduces the PEB, confirming the direct relationship between measurement diversity and localization accuracy. Remarkably, even with a single anchor, the inverse of the FIM remains well-defined, indicating that the localization problem remains identifiable and that all unknown parameters can, in principle, be estimated.

## 6. Conclusions and Future Directions

This paper presented a flexible, low-cost chipless RFID system for 3D self-localization and tracking in the THz band. The proposed architecture achieves sub-mm localization accuracy while minimizing reliance on fixed external infrastructure. The robot’s 3D position is estimated using hybrid measurements, namely RToF and AoA. To reduce the complexity of the estimation problem, RToF estimation is treated as a 1D problem via matched filtering, while a two-stage 2D MUSIC algorithm is employed to estimate AoA in azimuth and elevation. Specifically, the estimation is first conducted at the sub-UPA level, then the final estimates are obtained by combining the intermediate results through a simple averaging-based approach. Integrating this hybrid measurement approach with the self-localization and tracking framework significantly reduces dependence on fixed anchor nodes.

System performance was analyzed in terms of the PEB, highlighting the distinctions between snapshot localization PEB and Bayesian PEB. Enhanced measurements reduce snapshot localization PEB, whereas the spatial configuration of anchors strongly influences the rank of the FIM. In tracking scenarios, however, measurement accuracy alone is insufficient; the fidelity of the mobility model also plays a crucial role, with a more accurate model leading to better overall performance.

Numerical simulations at THz frequencies demonstrate that sub-mm localization and tracking are achievable even with a single anchor. With a reasonable number of antennas on the robot and only one connected tag, sub-mm accuracy can be obtained with 90% confidence. Similarly, in tracking scenarios, sub-mm accuracy is feasible using a single anchor, provided that sub-mm- to mm-level measurement accuracy or a sufficiently accurate mobility model can be provided, so the EKF can effectively correct the state estimate, whose performance depends directly on these two factors.

Finally, this paper highlights the potential of hybrid measurement schemes and motion-aware tracking algorithms to reduce infrastructure dependence while maintaining high-accuracy localization in dynamic environments.

The proposed framework offers a promising solution for next-generation high-accuracy self-localization and tracking applications. However, several practical implementation challenges may affect system performance under real-world operating conditions. These challenges include hardware impairments such as antenna channel phase mismatches, array calibration errors, phase noise, measurement synchronization inaccuracies, platform-induced vibrations, and orientation instability, particularly in aerial platforms. Such factors can degrade measurement quality and, consequently, localization and tracking accuracy.

Addressing these limitations is critical for achieving robust and reliable system operation in practical deployments. Therefore, future work will focus on systematically investigating the impact of these impairments and developing effective compensation and mitigation techniques. Experimental validation using real-world hardware platforms will be conducted to assess system performance under realistic conditions and to further enhance the robustness of the proposed approach.

## Figures and Tables

**Table 1:** Simulation Parameters.

Parameter	Value
Frequency range	220–330 GHz
Transmit power $P_{T}$	30 dBm
Number of epochs	100
Epoch duration *dt*	200 ms
Translational velocity *v*	1 m/s
Rotational velocity ω	$\frac{\pi }{10}$ rad/s
Room dimensions	10 m × 10 m × 3 m
Number/type of antenna array	25×25/square UPA
Number of Monte-Carlo runs	5000

## Data Availability

The data presented in this study are available on request from the corresponding author.

## Appendix A. Closed-Form Expression of J_*d*_(x)

Based on (10), and after some manipulations, the FIM for position estimation under independent distance observations is given by

(A1) $$\begin{array}{cc} J_{d}\left(x\right) & =\sum_{m=1}^{M}\frac{1}{{\left(\sigma_{d}^{\left(m\right)}\right)}^{2}}\frac{\left(x-p^{\left(m\right)}\right)}{∥x-p^{\left(m\right)}∥}\frac{{\left(x-p^{\left(m\right)}\right)}^{T}}{∥x-p^{\left(m\right)}∥}. \end{array}$$

Note that (A1) is very similar to (18), where each row of G is a unit vector pointing from the *m*th anchor to the target. For 2D positioning, the FIM in (10) becomes

(A2) $$\begin{array}{cc} J_{d-2D}\left(x\right)= & \sum_{m=1}^{M}\frac{1}{{\left(\sigma_{d}^{\left(m\right)}\right)}^{2}{∥x-p^{\left(m\right)}∥}^{2}}\times \\ \; & \left[\begin{array}{cc} {\left(x-p_{x}^{\left(m\right)}\right)}^{2} & \left(x-p_{x}^{\left(m\right)}\right)\left(y-p_{y}^{\left(m\right)}\right)\\ \left(x-p_{x}^{\left(m\right)}\right)\left(y-p_{y}^{\left(m\right)}\right) & {\left(y-p_{y}^{\left(m\right)}\right)}^{2} \end{array}\right]. \end{array}$$

Extending this result to 3D, the FIM can be written in closed form as

(A3) $$\begin{array}{cc} \; & J_{d-3D}\left(x\right)=\sum_{m=1}^{M}\frac{1}{{\left(\sigma_{d}^{\left(m\right)}\right)}^{2}{∥x-p^{\left(m\right)}∥}^{2}}\times \\ \; & \left[\begin{array}{ccc} {\left(x-p_{x}^{\left(m\right)}\right)}^{2} & \left(x-p_{x}^{\left(m\right)}\right)\left(y-p_{y}^{\left(m\right)}\right) & \left(x-p_{x}^{\left(m\right)}\right)\left(z-p_{z}^{\left(m\right)}\right)\\ \left(x-p_{x}^{\left(m\right)}\right)\left(y-p_{y}^{\left(m\right)}\right) & {\left(y-p_{y}^{\left(m\right)}\right)}^{2} & \left(y-p_{y}^{\left(m\right)}\right)\left(z-p_{z}^{\left(m\right)}\right)\\ \left(x-p_{x}^{\left(m\right)}\right)\left(z-p_{z}^{\left(m\right)}\right) & \left(y-p_{y}^{\left(m\right)}\right)\left(z-p_{z}^{\left(m\right)}\right) & {\left(z-p_{z}^{\left(m\right)}\right)}^{2} \end{array}\right], \end{array}$$

where ${\left(\sigma_{d}^{\left(m\right)}\right)}^{2}$ is the variance of the distance measurement error of the $m^{th}$ anchor.

## Appendix B. Closed-Form Expression of J_*θ*_(x)

Assume a 2D system; after some manipulations on (11), $J_{\theta }$ can be written as

(A4) $$\begin{array}{cc} J_{\theta -2D}\left(x\right)= & \sum_{m=1}^{M}\frac{1}{{\left(\sigma_{\theta }^{\left(m\right)}\right)}^{2}{∥x-p^{\left(m\right)}∥}^{4}}\times \\ \; & \left[\begin{array}{cc} {\left(y-p_{y}^{\left(m\right)}\right)}^{2} & -\left(x-p_{x}^{\left(m\right)}\right)\left(y-p_{y}^{\left(m\right)}\right)\\ -\left(x-p_{x}^{\left(m\right)}\right)\left(y-p_{y}^{\left(m\right)}\right) & {\left(x-p_{x}^{\left(m\right)}\right)}^{2} \end{array}\right]. \end{array}$$

Similarly, the FIM for 3D environment with AoA observations is $J_{\theta -3D}\left(x\right)$, and it is given by

(A5) $$\begin{array}{c} J_{\theta -3D}\left(x\right)=J_{\theta }\left(x\right)+J_{\phi }\left(x\right), \end{array}$$

where

(A6) $$\begin{array}{cc} J_{\theta }\left(x\right)= & \sum_{m=1}^{M}\frac{1}{{\left(\sigma_{\theta }^{\left(m\right)}\right)}^{2}{\left(D_{m}\right)}^{4}}\times \\ \; & \left[\begin{array}{ccc} {\left(y-p_{y}^{\left(m\right)}\right)}^{2} & -\left(x-p_{x}^{\left(m\right)}\right)\left(y-p_{y}^{\left(m\right)}\right) & 0\\ -\left(x-p_{x}^{\left(m\right)}\right)\left(y-p_{y}^{\left(m\right)}\right) & {\left(x-p_{x}^{\left(m\right)}\right)}^{2} & 0\\ 0 & 0 & 0 \end{array}\right], \end{array}$$

and

(A7) $$\begin{array}{cc} J_{\phi }\left(x\right)= & \sum_{m=1}^{M}\frac{1}{{\left(\sigma_{\phi }^{\left(m\right)}\right)}^{2}{∥x-p^{\left(m\right)}∥}^{4}}\times \\ \; & \left[\begin{array}{ccc} \frac{{\left(x-p_{x}^{\left(m\right)}\right)}^{2}{\left(z-p_{z}^{\left(m\right)}\right)}^{2}}{{\left(D_{m}\right)}^{2}}\; & \frac{\left(x-p_{x}^{\left(m\right)}\right)\left(y-p_{y}^{\left(m\right)}\right){\left(z-p_{z}^{\left(m\right)}\right)}^{2}}{{\left(D_{m}\right)}^{2}}\; & -\left(x-p_{x}^{\left(m\right)}\right)\left(z-p_{z}^{\left(m\right)}\right)\\ \frac{\left(x-p_{x}^{\left(m\right)}\right)\left(y-p_{y}^{\left(m\right)}\right){\left(z-p_{z}^{\left(m\right)}\right)}^{2}}{{\left(D_{m}\right)}^{2}}\; & \frac{{\left(y-p_{y}^{\left(m\right)}\right)}^{2}{\left(z-p_{z}^{\left(m\right)}\right)}^{2}}{{\left(D_{m}\right)}^{2}}\; & -\left(y-p_{y}^{\left(m\right)}\right)\left(z-p_{z}^{\left(m\right)}\right)\\ -\left(x-p_{x}^{\left(m\right)}\right)\left(z-p_{z}^{\left(m\right)}\right)\; & -\left(y-p_{y}^{\left(m\right)}\right)\left(z-p_{z}^{\left(m\right)}\right)\; & {\left(D_{m}\right)}^{2} \end{array}\right]. \end{array}$$

Here, $J_{\theta -3D}^{-1}\left(x\right)={\left(J_{\theta }\left(x\right)+J_{\phi }\left(x\right)\right)}^{-1}$, and since $J_{\theta }\left(x\right)$ in (A6) is singular, ${\mathrm{CRLB}}_{\theta }\neq {\mathrm{CRLB}}_{\theta }+{\mathrm{CRLB}}_{\phi }$. However, it is possible to estimate the location depending on the elevation angles only for less complexity at the expense of less accuracy and more required anchors, but this is not applicable with the azimuth angles.

It is interesting to note that $J_{d}\left(x\right)$, and $J_{\theta }\left(x\right)$ look very similar in structure except that $J_{d}\left(x\right)$ is inversely proportional to ${\left(\sigma_{d}^{\left(m\right)}\right)}^{2}{∥x-p^{\left(m\right)}∥}^{2}$, but $J_{\theta }\left(x\right)$ is inversely proportional to ${\left({\sigma_{\theta }}^{\left(m\right)}\right)}^{2}{∥x-p^{\left(m\right)}∥}^{4}$; this means that, in principle, $\sigma_{\theta }$ must be smaller by a factor of $∥x-p^{\left(m\right)}∥$ relative to $\sigma_{d}$ to produce the same FIM.

## Appendix C

**Proof of Proposition** **1.** If Y⪰0, then X+Y⪰X, and if X is nonsingular, this implies that:

(A8) $$\begin{array}{c} {\left(X+Y\right)}^{-1}⪯X^{-1}, \end{array}$$

and if Y is also nonsingular, then (A8) becomes

(A9) $$\begin{array}{c} {\left(X+Y\right)}^{-1}≺X^{-1}+Y^{-1}, \end{array}$$

Similarly, if X⪰0 and Y≻0, then

(A10) $$\begin{array}{c} {\left(X+Y\right)}^{-1}⪯Y^{-1}. \end{array}$$

By combining (A8) and (A10), X≻0 and Y≻0. Taking the trace yields

(A11) $$\begin{array}{c} \mathrm{tr}\left[{\left(X+Y\right)}^{-1}\right]<\min\left(\mathrm{tr}\left[X^{-1}\right],\mathrm{tr}\left[Y^{-1}\right]\right), \end{array}$$

which proves Proposition 1. □
